# Acute Aortic Dissection Type A in Younger Patients (< 60 Years Old) - Does Full Arch Replacement Provide Benefits Compared to Limited Approach?

**DOI:** 10.21470/1678-9741-2022-0434

**Published:** 2023-11-08

**Authors:** Ruslan Natanov, Malakh Lal Shrestha, Andreas Martens, Erik Beckmann, Heike Krueger, Morsi Arar, Linda Rudolph, Stefan Ruemke, Reza Poyanmehr, Wilhelm Korte, Tobias Schilling, Axel Haverich, Tim Kaufeld

**Affiliations:** 1 Department of Cardiothoracic, Transplantation and Vascular Surgery, Hannover Medical School, Hannover, Germany

**Keywords:** Cardiac Tamponade, Aortic Dissection, Morbidity, Artificial Respiration

## Abstract

**Introduction:**

Acute aortic dissection Stanford type A (AADA) is a surgical emergency associated with high morbidity and mortality. Although surgical management has improved, the optimal therapy is a matter of debate. Different surgical strategies have been proposed for patients under 60 years old. This paper evaluates the postoperative outcome and the need for secondary aortic operation after a limited surgical approach (proximal arch replacement) vs. extended arch repair.

**Methods:**

Between January 2000 and January 2018, 530 patients received surgical treatment for AADA at our hospital; 182 were under 60 years old and were enrolled in this study - Group A (n=68), limited arch repair (proximal arch replacement), and group B (n=114), extended arch repair (> proximal arch replacement).

**Results:**

More pericardial tamponade (P=0.005) and preoperative mechanical resuscitation (P=0.014) were seen in Group A. More need for renal replacement therapy (P=0.047) was seen in the full arch group. Mechanical ventilation time (P=0.022) and intensive care unit stay (P<0.001) were shorter in the limited repair group. Thirty-day mortality was comparable (P=0.117). New onset of postoperative stroke was comparable (Group A four patients [5.9%] vs. Group B 15 patients [13.2%]; P=0.120). Long-term follow-up did not differ significantly for secondary aortic surgery.

**Conclusion:**

Even though young patients received only limited arch repair, the outcome was comparable. Full-arch replacement was not beneficial in the long-time follow-up. A limited approach is justified in the cohort of young AADA patients. Exemptions, like known Marfan syndrome and the presence of an intimal tear in the arch, should be considered.

## INTRODUCTION

**Table:** 

Abbreviations, Acronyms & Symbols				
AADA	= Acute aortic dissection Stanford type A		GERAADA	= German Registry for Acute Aortic Dissection Type A
BMI	= Body mass index		HCA	= Hypothermic circulatory arrest
CABG	= Coronary artery bypass grafting		ICU	= Intensive care unit
CCT	= Cranial computed tomography		IQR	= Interquartile range
CI	= Confidence interval		IRAAD	= International Registry of Acute Aortic Dissection
COPD	= Chronic obstructive pulmonary disease		LCA	= Left coronary artery
CPB	= Cardiopulmonary bypass		PVOD	= Peripheral vascular occlusive disease
CT	= Computed tomography		RCA	= Right coronary artery
ECMO	= Extracorporeal membrane oxygenation		SACP	= Selective antegrade cerebral perfusion
ET	= Elephant trunk		SD	= Standard deviation
EVAR	= Endovascular aneurysm repair		TAA	= Thoraco-abdominal repair
FET	= Frozen elephant trunk		TEVAR	= Thoracic endovascular aortic repair

The development of an acute aortic dissection Stanford type A (AADA) is an emergency and should be surgically addressed as soon as possible. Despite surgical and technological advances, management of AADA is still challenging and is associated with relatively high morbidity and mortality^[1,2]^. In the acute setting, surgical resection of the intimal tear and replacement of the ascending aorta remain the golden standard for primary surgical management. However, due to the remaining dissection in the aortic arch, the possibility of aortic dilatation and rupture remains, and therefore, several groups have advocated for a more extensive aortic replacement. Two techniques have been proposed; the limited approach, where the ascending and proximal arches are replaced with prosthetic material, and the complete aortic arch replacement with either the elephant trunk (ET) or the frozen elephant trunk (FET) technique^[3,4]^. Both approaches have benefits, the limited aortic replacement is usually faster and requires less hypothermic circulatory arrest (HCA); the FET, however, replaces the complete arch and is beneficial for patients with extensive aortic disease and in need of secondary descending aortic surgery^[5]^. Although similar outcomes have been published for limited resection and full arch replacement, these results should be interpreted with caution as most data on full arch replacement comes from high-volume centers with extensive experience in aortic arch surgery^[6,7]^. Particularly in younger patients, the discussion of whether limited surgery is justified is of great importance, warranting data on long-term outcomes and the need for secondary aortic surgery^[8]^. To provide more evidence, we analyzed the outcome in our AADA population under 60 years old at the time of presentation after hemiarch surgery *vs.* full arch replacement.

## METHODS

### Study Population and Study Design

A retrospective analysis of all 503 patients receiving surgical treatment for AADA at our tertiary medical center between January 2000 and January 2018 was done. De Bakey II dissections were not included in this study. Over one-third of all patients (182 patients; 36.2%) were under 60 years old at the time of presentation. The mean patient age of the under 60-year cohort was 51.3 years (interquartile range [IQR] 45.4 - 56.1 years). The study population was divided into two groups: patients treated with a limited approach including replacement of the ascending aorta and proximal arch (n=68; 37.4%) and patients treated with complete arch replacement (> prox. arch) (n=114; 62.6%). All data were collected retrospectively and were approved by our institutional ethics committee (10519_BO_K_2022). All patients’ characteristics are stated in Table 1. Follow-up of patient data ended on 01/2022 and was 100%.

**Table 1:** Patients’ characteristics.

Characteristics	Patients ≤ 60 years	Prox. arch replacement	Extended arch repair	*P*-value
Total of patients <60 years old	n=182	n=68	n=114	
Cerebral malperfusion, n (%)	18 (9.9)	8 (11.8)	10 (8.8)	0.324
Visceral malperfusion, n (%)	16 (8.8)	6 (8.8)	10 (8.8)	0.991
Limb malperfusion, n (%)	32 (17.6)	9 (13.2)	23 (20.2)	0.234
Renal malperfusion, n (%)	25 (13.7)	6 (8.8)	19 (16.7)	0.137
Hemiparesis, n (%)	10 (5.5)	5 (7.4)	5 (4.4)	0.504
Paraparesis, n (%)	7 (3.8)	3 (4.4)	4 (3.5)	1.000
Seizure, n (%)	1 (0.5)	0 (0.0)	1 (0.9)	1.000
Evidence of stroke CT, n (%)	10 (5.5)	4 (5.9)	6 (5.3)	1.000
Neurologic symptoms, n (%)	31 (17.0)	14 (20.6)	17 (14.9)	0.324
Dissection of supra-aortic arteries, n (%)	36 (19.8)	13 (19.1)	23 (20.2)	0.862
Dissection of LCA, n (%)	5 (2.7)	0 (0.0)	5 (4.4)	0.159
Dissection of RCA, n (%)	19 (10.4)	8 (11.8)	11 (9.6)	0.652
Iatrogenic dissection, n (%)	1 (0.5)	0 (0.0)	1 (0.9)	1.000
Painful event prior to surgery (hours), median (IQR)	7.0 (4.0 - 18.0)	6.0 (4.0 - 15.0)	7.0 (4.0 - 21.3)	0.577

CT=computed tomography; IQR=interquartile range; LCA=left coronary artery; RCA=right coronary artery

### Definitions

Patients with AADA may either present specific symptoms, like floating thoracic and lumbar pain, abdominal pain, signs of malperfusion, and neurological disabilities, or unspecific symptoms. Finding of an intimal tear, intramural hematoma, or a dissection membrane using multi-slice computed tomography (CT) was mandatory for the diagnosis of AADA. Arterial occlusion or false lumen perfusion has been defined according to Sievers et al. (“type, entry, malperfusion” [or TEM] Classification, stages M2 and M3 [-], [+]) as malperfusion^[9]^. Patients who presented severe neurological symptoms like hemiplegia, apraxia, or dysarthria without performing cerebral CT prior to surgery were assigned to the preoperative stroke cohort. Cerebral stroke had to be verified using CT magnetic resonance imaging. AADA accidentally induced during open-heart surgery was defined as iatrogenic dissection. Because preoperative transesophageal echocardiography was not frequently performed in AADA patients, pericardial tamponade was defined as a bloody pericardial effusion > 1 cm using CT. According to our standardized operating procedure, a postoperative control CT scan was performed on all patients. Postoperatively detected malperfusion was defined as persisting malperfusion.

### Perioperative Management and Surgical Technique

According to our standardized protocol, all patients with an acute AADA are promptly transferred to the operation theatre after confirmation of the diagnosis. To avoid early decompensation, intubation was not performed before complete preoperative preparation. After intubation, a median sternotomy and central cannulation for extracorporeal circulation were established. Central cannulation was done as previously described^[9,8]^. In brief, a guidewire was placed in the true lumen under transesophageal echocardiographic control. Subsequently, the cannulation of the ascending aorta was done with the Seldinger’s technique. Due to the long period covered by this study, the surgical technique regarding the choice of aortic grafts evolved significantly.

**Fig. 1:**
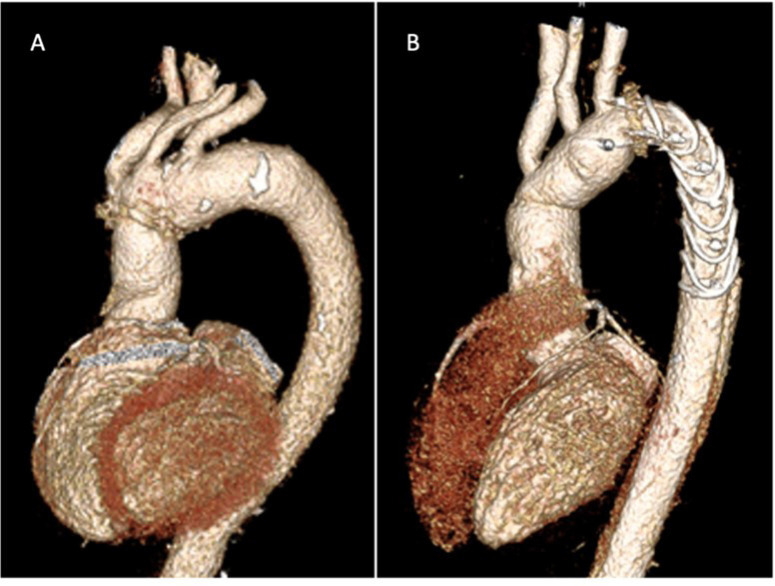
Surgical treatment of acute aortic dissection Stanford type A. A) Proximal arch replacement; B) total arch replacement (frozen elephant trunk).

During the period from 2000 to 2010, the FET technique was performed using the custom-made Chavan-Haverich prosthesis followed by the prefabricated Chavan-Haverich hybrid graft (Curative GmbH, Dresden, Germany). The use of the Jotec E-vita® hybrid graft was established after it became available. Until 2010, the island technique was performed to reattach the supra-aortic vessels. In cooperation with Vascutek Terumo (Terumo®, Glasgow, United Kingdom), we developed the four-branched FET which was frequently used since 2010. For a total or hemiarch replacement, we changed our strategy from a straight graft with island technique to the branched Sienna™ graft (Terumo®, Glasgow, United Kingdom) in 2008. Due to the extensive use of branched aortic arch, prosthesis resulted in major technical changes. As a consequence of these changes, the arch replacement was performed after completing the cardiac and distal aortic repair. Head vessels were anastomosed to the corresponding side branches of the graft at the end of the procedure. In all cases, either a proximal, subtotal (involving replacement of the brachiocephalic trunk), or total arch replacement with ET or FET, HCA (temperatures between 22°C and 26°C), and bilateral selective antegrade cerebral perfusion (SACP) were performed. In 2010, we started the beating heart technique for cardioprotection during total arch repair. An isolated replacement of the proximal aortic arch was performed using a straight Dacron® graft or a Gelweave™ Ante-Flo beginning in 2010. CT imaging of the proximal arch and the four-branched FET is shown in Figure 1.

### Statistical Analysis

SPSS 27 Statistics software (IBM Corp. Released 2020; IBM SPSS Statistics for Windows, Version 27.0; Armonk, NY: IBM Corp.) was used for data analysis. Normal distribution of variables was analyzed with the Kolmogorov-Smirnov test. Categorical variables are stated as absolute numbers (n) and proportions. Normally distributed continuous variables are stated as mean ± standard deviation, while continuous variables without normal distribution are stated as the median and IQR. Chi-square test, Fisher’s exact test, Mann-Whitney U test, and *t*-test were used to detect differences between the groups. Kaplan-Meier analysis was applied for the evaluation of survival, and the log-rank test was used to test for differences. We did not correct for multiple testing. *P*-value < 0.05 was considered as statistically significant.

## RESULTS

During the study period, 503 patients were surgically treated for AADA in our tertiary hospital. Of the total population, the subgroup of patients younger than 60 years old at the time of presentation consisted of 182 (36.2%) patients. The median patient age was 51.3 years (group A 51.5 years [46.5-57.6] *vs.* group B 50.9 years [44.3-55.6]; *P*=0.223). The population was predominantly male (group A 79.4% [n=54] *vs.* group B 82.5% [n=94]; *P*=0.610) and had a median body mass index of 26.9 (group A 27.4 [24.8-30.8] *vs.* group B [24.6-29.3]; *P*=0.291). Arterial hypertension (group A 55.9% [n=38] *vs.* group B 69.3% [n=79]; *P*=0.068) and chronic obstructive pulmonary disease (group A 4.4% [n=3] *vs.* group B 9.6% [n=11]; *P*=0.200) did not occur significantly more often in group B. Coronary artery disease (group A 13.2% [n=9] *vs.* group B 6.1% [n=7]; *P*=0.102) and diabetes mellitus (group A 8.8% [n=6] *vs.* group B 1.8% [n=2]; *P*=0.054) were less commonly present in group B. Marfan syndrome was seen in 18 patients (9.9%); most of Marfan patients underwent extended arch surgery (group A 4.4% [n=3] *vs.* group B 13.2% [n=15]; *P*=0.056). Significant differences were detected regarding preoperative conditions like pericardial tamponade (group A 48.5% [n=33] *vs.* group B 28.1% [n=32]; *P*=0.005) and preoperative mechanical resuscitation (group A 14.7% [n=10] *vs.* group B 4.4% [n=5]; *P*=0.014). Other patients’ characteristics were equally distributed and are stated in Table 1.

Preoperative data are shown in Table 2. Preoperative signs of malperfusion were seen in 66 patients (33.5%). Further preoperative data were comparable between both groups - stroke (group A 5.9% [n=4] *vs.* group B 5.3% [n=6]; *P*=1.000) and dissection of supra-aortic arteries (group A 19.1% [n=13] *vs.* group B 20.2% [n=23]; *P*=0.862).

**Table 2:** Preoperative data.

Characteristics	Patients ≤ 60 years	Prox. arch replacement	Extended arch repair	*P*-value
Total of patients <60 years old	n=182	n=68	n=114	
Age at surgery (years), median (IQR)	51.3 (45.4 - 56.1)	51.5 (46.5 - 57.6)	50.9 (44.3 - 55.6)	0.223
Sex, male, n (%)	148 (81.3)	54 (79.4)	94 (82.5)	0.610
BMI, median (IQR)	26.9 (24.7 - 30.1)	27.4 (24.8 - 30.8)	26.7 (24.6 - 29.3)	0.291
Hypertension, n (%)	117 (64.3)	38 (55.9)	79 (69.3)	0.068
Diabetes mellitus, n (%)	8 (4.4)	6 (8.8)	2 (1.8)	0.054
PVOD, n (%)	6 (3.3)	3 (4.4)	3 (2.6)	0.673
COPD, n (%)	14 (7.7)	3 (4.4)	11 (9.6)	0.200
Coronary heart disease, n (%)	16 (8.8)	9 (13.2)	7 (6.1)	0.102
Hyperthyroid, n (%)	0 (0.0)	0 (0)	0 (0)	-
Hypothyroid, n (%)	13 (7.1)	7 (10.3)	6 (5.3)	0.240
Atrial fibrillation, n (%)	12 (6.6)	5 (7.4)	7 (6.1)	0.765
Marfan syndrome, n (%)	18 (9.9)	3 (4.4)	15 (13.2)	0.056
Bicuspid aortic valve, n (%)	11 (6.0)	3 (4.4)	8 (7.0)	0.541
Pericardial tamponade, n (%)	65 (35.7)	33 (48.5)	32 (28.1)	0.005
Preoperative intubation, n (%)	27 (14.8)	13 (19.1)	14 (12.3)	0.209
Mechanical resuscitation, n (%)	15 (8.2)	10 (14.7)	5 (4.4)	0.014
Cardiac reoperation, n (%)	5 (2.7)	2 (2.9)	3 (2.6)	1.000
Malperfusion, n (%)	61 (33.5)	22 (32.4)	39 (34.2)	0.797

BMI=body mass index; COPD=chronic obstructive pulmonary disease; IQR=interquartile range; PVOD=peripheral vascular occlusive disease

Intraoperative data showed a significantly lower total operation time (group A 294.9 min ± 81.5 *vs.* group B 395.5 min ± 91.4; *P*<0.001), cardiopulmonary bypass (CPB) time (group A 191.2 min ± 59.2 *vs.* group B 280 min ± 75.2; *P*<0.001), and aortic cross-clamping time (group A 116.9±40.2 *vs.* group B 160.5±51.9; *P*<0.001) in the limited arch repair group. Furthermore, the median time needed for HCA (group A 26.5 min [21.0-35.0] *vs.* group B 52.0 min [37.8-70]; *P*<0.001) and median SACP time (group A 20.0 min [16.3-27.8] *vs.* group B 74 min [47.8-95.3]; *P*<0.001) were significantly shorter in the proximal arch population. More patients were treated with the beating heart technique in the full arch population (group A 2.9% [n=2] *vs.* group B 27.2% [n= 31]; *P*<0.001). Aortic root involvement was seen equally in both populations. However, the Bentall procedure for root replacement was done significantly more in the proximal arch population (group A 38.2% [n=26] *vs.* group B 23.7% [n=27]; *P*=0.037) in comparison to the full arch population. Interestingly, aortic valve reconstruction (David operation) was significantly favored in group B (group A 23.5% [n=16] *vs.* group B 41.2% [n=47]; *P*=0.015). Other intraoperative characteristics did not differ significantly and are stated in Table 3.

**Table 3:** Intraoperative data.

Characteristics	Patients ≤ 60 years	Prox. Arch replacement	Extended arch repair	*P*-value
Total of patients <60 years old	n=182	n=68	n=114	
Total operation time (min), mean ± SD	358.2±100.4	294.9±81.5	395.9±91.4	< .001
Cardiopulmonary bypass time (min), mean ± SD	246.8±81.8	191.2±59.3	280.0±75.2	< .001
Aortic cross-clamping time (min), mean ± SD	144.2±52.2	116.9±40.2	160.5±51.9	< .001
Hypothermic circulatory arrest time (min), median (IQR)	40.5 (26.8 - 61.0)	26.5 (21.0 - 35.0)	52.0 (37.8 - 70.0)	< .001
Selective antegrade cerebral perfusion time (min), median (IQR)	47.0 (22.0 - 84.3)	20.0 (16.3 - 27.8)	74.0 (47.0 - 95.3)	< .001
Minimal core temperature (°C), median (IQR)	24.4 (21.5 - 26.0)	25.0 (21.0 - 26.1)	24.0 (21.5 - 25.2)	0.115
Erythrocyte concentrates, median (IQR)	6.0 (3.0 - 9.0)	5.5 (3.0 - 9.0)	6.0 (3.0 - 9.3)	0.458
Fresh frozen plasma, median (IQR)	6.0 (4.0 - 10.0)	6.0 (5.0 - 10.0)	6.0 (4.0 - 10.0)	0.746
Platelet concentrates, median (IQR)	3.0 (2.0 - 4.0)	2.0 (2.0 - 4.0)	3.0 (2.0 - 4.0)	0.093
Beating heart, n (%)	33 (18.1)	2 (2.9)	31 (27.2)	< .001
Proximal arch replacement, n (%)	68 (37.4)	68 (100.0)	0 (0.0)	
Subtotal arch replacement, n (%)	7 (3.8)	0 (0.0)	7 (6.1)	0.047
Total arch replacement, n (%)	13 (7.1)	0 (0.0)	13 (11.4)	0.002
Total arch replacement, elephant trunk, n (%)	24 (13.2)	0 (0.0)	24 (21.1)	< .001
Total arch replacement, frozen elephant trunk, n (%)	70 (38.5)	0 (0.0)	70 (61.4)	< .001
BioGlue®, n (%)	46 (25.3)	14 (20.6)	32 (28.1)	0.261
Aortic valve replacement, biological, n (%)	11 (6.0)	5 (7.4)	6 (5.3)	0.749
Aortic valve replacement, mechanical, n (%)	42 (23.1)	21 (30.9)	21 (18.4)	0.054
Root involvement, n (%)	128 (70.3)	47 (69.1)	81 (71.1)	0.782
Bentall procedure, n (%)	53 (29.1)	26 (38.2)	27 (23.7)	0.037
David procedure, n (%)	63 (34.6)	16 (23.5)	47 (41.2)	0.015
Yacoub procedure, n (%)	10 (5.5)	5 (7.4)	5 (4.4)	0.504
CABG, n {%)	31 (17.0)	11 (16.2)	20 (17.5)	0.812
ECMO, n (%)	9 (4.9)	2 (2.9)	7 (6.1)	0.487
Exitus in tabula, n (%)	4 (2.2)	2 (2.9)	2 (1.8)	0.630

CABG=coronary artery bypass grafting; ECMO=extracorporeal membrane oxygenation; IQR=interquartile range; SD=standard deviation

### Postoperative Outcome and Survival

**Fig. 2:**
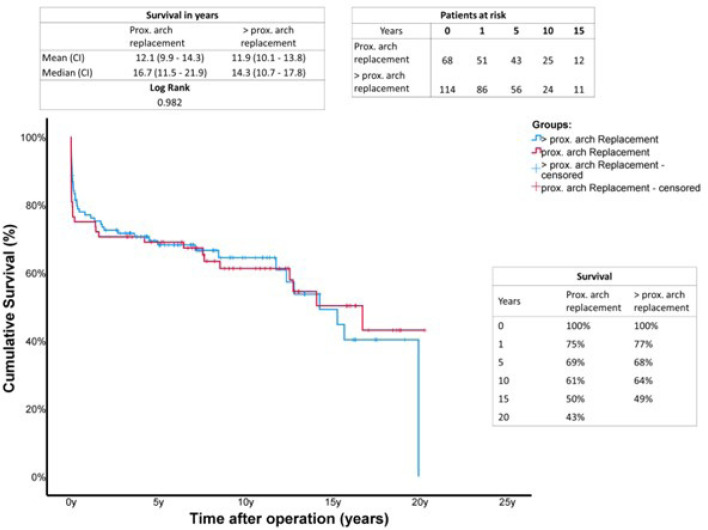
Kaplan-Meier curves showing survival with limited (proximal arch) and extended (> proximal arch) aortic repair. The x-axis denotes the time after operation. CI=confidence interval.

The postoperative outcome is shown in Table 4. Early survival (30-day mortality) was equal in both populations. The proximal arch population was on mechanical ventilation for a significantly shorter time (group A 32 hours [115.3-87.8] *vs.* group B 55 hours [21.5-179.5]; *P*=0.022) and had shorter patient stay in the intensive care unit (ICU) (group A 3.0 days [2.0-5.0] *vs.* group B 5.0 days [3.0-9.0]; *P*<0.001). Furthermore, more renal failure with temporary postoperative need for dialysis was seen in the full arch replacement population (group A 5.9% [n=4] *vs.* group B 15.8% [n=18]; *P*=0.047). Newly diagnosed strokes using multi-slice CT were equal in the limited approach (four patients; 5.9%) and the extended arch surgery (15 patients; 13.2%) populations. Follow-up data are displayed in Table 5. The surviving population showed no significant difference in the rate of secondary aortic operations, and reoperation of the aorta in the identic area or downstream aorta. Furthermore, the rate of thoracoabdominal aortic repair was similar in both groups. Kaplan-Meyer (Figure 2) analysis for survival after a 20-year follow-up showed no significant benefit for either population.

**Table 4:** Postoperative data.

Characteristics	Patients ≤ 60 years	Prox. arch replacement	Extended arch repair	*P*-value
Total of patients <60 years old	n=182	n=68	n=114	
Survival time (days), median (IQR)	2156.5 (380.0 - 4008.0)	2765.5 (181.3 - 4631.5)	1779.0 (380.0 - 3325.5)	0.138
Ventilation time (hours), median (IQR)	45.5 (18.0 - 139.0)	32.0 (15.3 - 87.8)	55.0 (21.5 - 179.5)	0.022
Intensive care unit (days), median (IQR)	4.0 (2.0 - 8.0)	3.0 (2.0 - 5.0)	5.0 (3.0 - 9.0)	< .001
Rethoracotomy, n (%)	35 (19.2)	10 (14.7)	25 (21.9)	0.232
Dialysis, n (%)	22 (12.1)	4 (5.9)	18 (15.8)	0.047
30-day mortality, n (%)	30 (16.5)	15 (22.1)	15 (13.2)	0.117
CCT stroke, n (%)	36 (19.8)	9 (13.2)	27 (23.7)	0.087
New-onset stroke, n (%)	19 (10.4)	4 (5.9)	15 (13.2)	0.120
Paraparesis, n (%)	8 (4.4)	4 (5.9)	4 (3.5)	0.474
Hemiparesis, n (%)	6 (3.3)	1 (1.5)	5 (4.4)	0.413
Persistent cerebral malperfusion, n (%)	7 (3.8)	1 (1.5)	6 (5.3)	0.260
Persistent limb malperfusion, n (%)	3 (1.6)	2 (2.9)	1 (0.9)	0.557
Persistent visceral malperfusion, n (%)	5 (2.7)	1 (1.5)	4 (3.5)	0.652
Persistent renal malperfusion, n (%)	8 (4.4)	4 (5.9)	4 (3.5)	0.474

CCT=cranial computed tomography; IQR=interquartile range

**Table 5:** Follow-up data.

Characteristics	Patients ≤ 60 years	Prox. arch replacement	Extended arch repair	*P*-value
Total of patients <60 years old	n=182	n=68	n=114	
Secondary aortic operation, n (%)	34 (18.7)	12 (17.6)	22 (19.3)	0.782
Reoperation of identic area, n (%)	10 (5.5)	4 (5.9)	6 (5.3)	1.000
Reoperation of downstream aorta, n (%)	24 (13.2)	8 (11.8)	16 (14.0)	0.661
TAA repair, n (%)	7 (3.8)	2 (2.9)	5 (4.4)	1.000
Y-prothesis, n (%)	2 (1.1)	1 (1.5)	1 (0.9)	1.000
Descending repair, n (%)	14 (7.7)	4 (5.9)	10 (8.8)	0.479
Hybrid, n (%)	5 (2.7)	3 (4.4)	2 (1.8)	0.364
TEVAR, n (%)	7 (3.8)	1 (1.5)	6 (5.3)	0.260
EVAR, n (%)	2 (1.1)	1 (1.5)	1 (0.9)	1.000
Aortic fenestration	0 (0.0)	0 (0.0)	0 (0.0)	-

EVAR=endovascular aneurysm repair; TAA=thoraco-abdominal repair; TEVAR=thoracic endovascular aortic repair

## DISCUSSION

In this paper, we examined the difference in postoperative outcome and long-term follow-up between a limited approach, *i.e.*, replacement of the ascending aorta and proximal arch, and an extended arch repair in patients under 60 years old at the time of admission for AADA. Although our findings are from a single center and thus pose a major limitation, the number of patients included justifies this study. Our results showed almost similar preoperative patient characteristics in both groups. There were significantly more patients with pericardial tamponade and mechanical resuscitation in the hemiarch group. Previous data show a clear negative association between preoperative pericardial tamponade and patient outcome^[11]^. Taking this into consideration, a limited approach is warranted in these patients to assure intraoperative survival. This might explain the higher incidence of pericardial tamponade in the hemiarch group. When compared to the data from the German Registry for Acute Aortic Dissection Type A (GERAADA), our population showed fewer rates of pericardial tamponade and the need for resuscitation^[12]^. This may play a role in the decision to refrain from full arch surgery. Patients presented in reduced conditions usually receive the shortest operation to enable primary patient survival. However, data from the GERAADA includes patients of all ages, and the comparison should be made with caution^[13]^. Intraoperative data showed a significant difference in HCA and SACP and CPB time favoring the hemiarch population, similar results were seen in the data of the International Registry of Acute Aortic Dissection (IRAAD)^[14]^. Furthermore, the extended arch repair cohort developed significantly more renal failure with the need for dialysis, this was also seen in previous studies^[15]^. This may be attributed to the longer HCA and CPB times; previous research has shown a relationship between longer HCA and renal failure, interestingly no significant relation between time on CPB and renal failure was seen in AADA patients^[16]^. Intraoperative results showed more aortic root replacements in the hemiarch population, whereas more aortic valve-sparing procedures were performed in the full arch population. For the Bentall procedure, similar results were published by others^[6]^. The valve-sparing root procedure, however, although feasible and safe is a matter of debate. Although the procedure is feasible and, when performed correctly, does not impair postoperative outcome^[17,18]^, it should be performed by experienced surgeons. In high-volume centers with great experience, similar results may be achieved. This is however not the standard therapy of choice and some centers have reported poor durability of the aortic valve^[14,19,20]^. Therefore, it should not be advocated in all cases. Postoperative data showed significantly longer ICU stay and mechanical ventilation time in the full arch replacement group. However, besides the previously mentioned higher rate of renal replacement therapy in the full arch group, the complication rate was not significantly different. Furthermore, overall survival did not differ between our populations, and both the IRAAD and GERAADA registries support these findings^[13,14]^. Long-time follow-up data from our patient population showed no difference in the rate for secondary aortic surgery and reoperation of the identic area of the downstream aorta. This data supports the notion of the limited approach in the acute setting. Data on the long-term effects of limited *vs.* full arch repair are scarce, however, one study found similar results in the rate of reoperation. Again, patients of all ages were included in this study and, therefore, should be compared with caution^[6]^.

## CONCLUSION

Surgical management of the patient presenting with AADA can be difficult and daunting. The decision between a limited approach and full arch replacement is difficult, especially in younger patients. Though full arch replacement results have improved over the last decades, this type of operation belongs to the realm of experienced centers and surgeons. Even though patients treated with a limited approach were in significantly poorer condition, our data have shown comparable complication rates and survival in patients treated with a limited arch repair. The use of FET is a viable option, especially in young patients with the presence of malperfusion, patients with Marfan syndrome, and the presence of an intimal tear in the arch. A limited approach is particularly beneficial in young and compromised patients. We conclude that a limited approach is a feasible option for surgeons and clinics with limited experience in the field of acute aortic surgery.
